# Can MRI Accurately Diagnose and Stage Endometrial Adenocarcinoma?

**DOI:** 10.3390/medicina60030512

**Published:** 2024-03-21

**Authors:** Ramona-Andreea Rizescu, Iulia Alecsandra Sălcianu, Alexandru Șerbănoiu, Radu Tudor Ion, Lucian Mihai Florescu, Ioana-Andreea Gheonea, Gheorghe Iana, Ana Magdalena Bratu

**Affiliations:** 1 Doctoral School of the University of Medicine and Pharmacy Craiova, 200349 Craiova, Romania; ramona.rizescu11@gmail.com; 2 Department of Radiology and Medical Imaging, University of Medicine and Pharmacy Carol Davila, 050474 Bucharest, Romania; radu.ion@drd.umfcd.ro (R.T.I.); george_iana@yahoo.com (G.I.); ana.bratu@umfcd.ro (A.M.B.); 3 Department of Radiology and Medical Imaging, Colțea Hospital, 030171 Bucharest, Romania; 4 Department of Radiology and Medical Imaging, University Emergency Hospital Bucharest, 050098 Bucharest, Romania; 5 Doctoral School of “Carol Davila”, University of Medicine and Pharmacy, 700115 Bucharest, Romania; 6 Department of Radiology and Medical Imaging, University of Medicine and Pharmacy Craiova, 200349 Craiova, Romania; lucian.florescu@umfcv.ro (L.M.F.); ioana.gheonea@umfcv.ro (I.-A.G.)

**Keywords:** endometrial cancer, endometroid adenocarcinoma, Federation of Gynecology and Obstetrics staging, magnetic resonance imaging staging

## Abstract

*Background and Objectives*: Endometrial carcinoma is one of the most common gynecological cancers, and benign lesions such as endometrial hyperplasia, polyps, adenomyosis and leiomyomas should be included in the differential diagnosis. Magnetic resonance imaging has an important role in evaluating endometrial cancer and assessing the depth of myometrial invasion, and it closely correlates with the prognosis of the patient. The purpose of this study is to evaluate the MRI semiology of the endometrial carcinomas that mimic benign lesions, the main factors that may affect the correct diagnosis and the feasibility of magnetic resonance imaging to evaluate the depth of the myometrial invasion of endometrial cancer. *Materials and Methods*: This is a retrospective analysis of 45 patients that underwent MRI examinations and the lesions were pathologically diagnosed as endometrial carcinoma after surgical resection. This study evaluated the staging accuracy of T2-weighted imaging, diffusion-weighted imaging (DWI), ADC mapping and T1-weighted imaging with fat saturation before and after gadolinium injection. *Results*: In 36 of the 45 cases, the MRI of the lesion showed the characteristics of endometrial cancer and the diagnosis was certain. Nine lesions (20%) were described as unequivocal and had unspecific MR appearance. In eight of the nine cases (89%), the histopathologic report revealed the presence of leiomyomas and two of these cases (22%) were also associated with adenomyosis. The cause of underestimation in these patients was coexisting lesions exhibiting heterogenous intensity and contrast enhancement, which made it difficult to detect the margins of the lesions. The depth of the myometrial invasion was underestimated in nine cases and overestimated in three cases. The staging accuracy with MRI was 74%. There was a significant correlation between MR imaging and histopathologic finding in the assessment of myometrial invasion (*p* < 0.001). Cervical extension was noted in eight cases (18%), but was missed on MR imaging in two patients and overstaged in none. Six of them were associated with myometrial invasion in more than 50% of the thickness. There was a significant correlation between MR imaging and histopathologic finding in the assessment of cervical extension (*p* < 0.001). *Conclusions*: Our data confirm the high accuracy of MRI in the diagnosis and local staging of endometrial carcinoma. The information provided by MRI has an important role in planning the treatment and the prognosis of the patients.

## 1. Introduction

Endometrial carcinoma has an incidence rate that accounts for more than 50% malignant tumors in female patients, being one of the most common gynecological cancers [1]. The histological type, tumor grade and the International Federation of Obstetrics and Gynecology (FIGO 2023) staging have a great impact on the prognosis and treatment of endometrial cancer.

Magnetic resonance imaging (MRI) is widely applied in the diagnosing and staging of endometrial cancer [2]. MRI is the best tool for assessing myometrial invasion depth and cervical extension, which correspond with the grade of the tumor, the presence of lymph node invasion and prognosis [3,4,5]. Conventional MRI is based on the radiologist’s perception and experience and sometimes presents interobserver variations [6,7].

This study attempts to comprehensively explore the information derived from T2-weighted imaging, diffusion-weighted imaging (DWI), ADC mapping and T1-weighted imaging with fat saturation before and after gadolinium injection in order to preoperatively distinguish endometrial carcinoma from benign mimics.

Local staging of endometrial cancer requires evaluating the tumor extension in the thickness of the myometrium. Observing an intact junctional zone, with low signal intensity on T2-weighted images and a thin band of early subendometrial enhancement excludes almost completely myometrial invasion [4,5]. The disruption of the subendometrial band indicates myometrial invasion. If there is an invasion of less than 50% of the myometrial thickness, the staging of the tumor is IA, while invasion greater than or equal to 50% of the myometrial thickness indicates a stage IB tumor [8].

There are some possible pitfalls that can result in underestimation and overestimation of myometrial invasion: (1) the tumor may present as a small or isointense lesion; (2) there may be poor visualization of the endometrium or the endometrial–myometrial interface contrast due to the presence of leiomyomas, adenomyosis, myometrium thinning in postmenopausal patients or endometrial thinning in older patients or endometrial cavity distension [4,5].

The purpose of this study is to evaluate the accuracy of MR imaging in diagnosing and staging endometrial carcinoma based on histopathological findings. Another aim is to identify the benign lesions that may affect the correct staging of the tumor.

## 2. Materials and Methods

### 2.1. Patient Selection

A retrospective review of the oncologic database from our institution, from January 2019 to November 2023, included 45 women, aged 34 to 81 years (age mean 60 years), who presented with abnormal vaginal bleeding and had undergone MRI examinations. Thirty-five of the patients (78%) attained menopause at the time of presentation. All lesions were pathologically diagnosed with endometrial carcinoma after surgical resection. Four patients were excluded from this study based on the definitive histologic diagnosis that differed from adenocarcinoma: carcinosarcoma in one patient and leiomyosarcoma in three patients.

Our institutional review board does not require approval for this type of retrospective study.

The clinical, pathologic and imaging parameters were collected from electronic medical records.

Patient data are compiled and stored in a anonymized and centralized Microsoft Excel© spreadsheet. Data analysis and graphical representations were performed using SPSS Statistics software (version 29.0 IBM Corporation, Armonk, NY, USA) and Microsoft Excel© (version 16.83, Microsoft, Redmond, WA, USA). The analysis of the correlation between MRI findings and histopathological results was performed using the independent-samples *t*-test. The statistical significance for the test was chosen to be *p* < 0.05.

### 2.2. MRI Protocol

The MRI scan was performed with patients in supine position, with free breathing during acquisition on 1.5 T scanners with phased-array abdominal coils.

The protocol included high-resolution sagittal T2-weighted fast spin-echo imaging, T2-weighted scans coronally and axially to the longitudinal axis of the uterine body, diffusion-weighted imaging (DWI) with b = 0 and 800 s/mm^2^ (the ADC map is automatically calculated based on DWI by the embedded software of the MRI equipment), T1-weighted images with fat suppression before and after intravenous administration of 10 mL of gadolinium followed by flushing with 20 mL saline. 

### 2.3. Image Analysis

There are several signal characteristics of endometrial cancer on MRI that are commonly recognized. In T2-weighted images, the lesion appears as a well-delineated (regular or irregular) or diffuse mass and is located within the endometrial cavity, showing heterogenous intermediate signal intensity relative to the hyperintense endometrium and hypointense myometrium. In diffusion-weighted images, the tumor appears hyperintense at the high B value, with a corresponding hypointense signal on the ADC map. On the postcontrast images, tumors may show early uptake in comparison with the normal endometrium and slower uptake than the myometrium. The proper timing for evaluating myometrial invasion is 2.5 min after contrast injection. Images obtained at 4 min after contrast injection are useful in detecting cervical invasion [3,4,5,9,10].

## 3. Results

**Figure 1:**
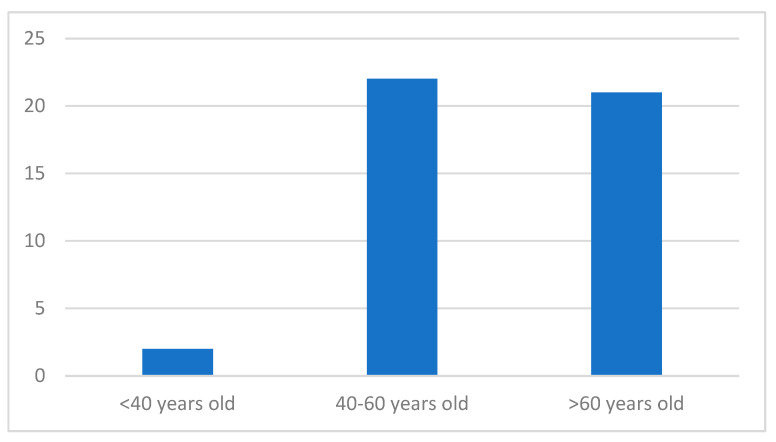
Age distribution of patients included in this study.

**Figure 2:**
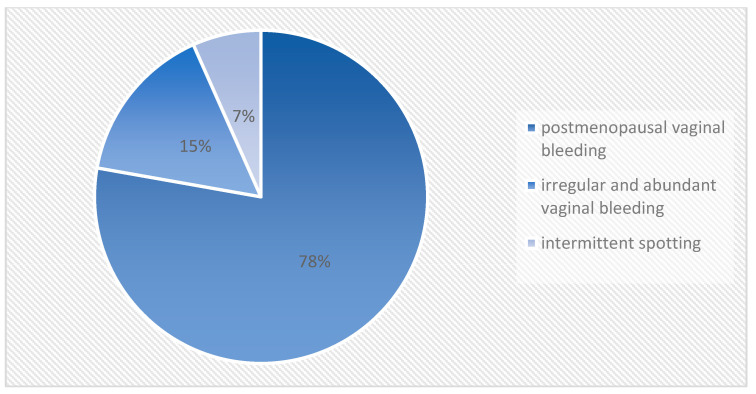
Presenting symptoms of the patients included in this study.

Of the patients, 22 (49%) were in the age group between 41 and 60 years, followed by 21 patients (47%) who were more than 60 years old and 2 patients who were less than 40 years old (4%) (Figure 1). The most common symptom was postmenopausal vaginal bleeding, observed in 35 patients (78%). Patients in the perimenopausal age group had irregular and abundant vaginal bleeding (7 patients, 15%), but also intermittent spotting (3 patients, 7%) (Figure 2).

On T2-weighted images, endometrial carcinoma appeared hypointense to the adjacent myometrium in 16 patients (35%), isointense in 22 patients (49%) and hyperintense in 7 patients (16%). Forty of the tumors showed high signal intensity on DWI (89%). The contrast enhancement of the tumors was assessed as low in 30 cases (67%), intermediate in 3 cases (7%) and heterogenous in 12 cases (26%). The endometrium appeared thickened in 21 cases (46%).

The maximum diameter of the tumor was evaluated; in 7 cases (16%) it measured maximum 20 mm, 21 to 40 mm in 24 cases (53%), 41 to 60 mm in 10 cases (22%), and over 60 mm in 4 cases (9%).

Of these 45 cases of endometrial cancer, 38 patients had endometrioid adenocarcinomas (15 were well differentiated: grade G1; 18 had moderate differentiation: grade G2; 5 were poorly differentiated: grade G3), 4 women had serous adenocarcinoma, 1 had clear cell adenocarcinoma, and 2 cancers were mixed adenocarcinoma (clear cell and serous adenocarcinoma). All of the patients with endometrial carcinoma underwent standard surgical procedure, total abdominal hysterectomy with bilateral salphingo-oophorectomy with pelvic lymph node dissection, and the staging accuracies of the T2, DWI and gadolinium-enhanced T1-weighted images in the assessment of myometrial invasion were evaluated.

The findings on MR imaging were compared with surgical and pathological findings.

Histological examination of the 45 endometrial cancers revealed that tumors were confined to the endometrium in 3 cases, invaded less than 50% of the myometrial thickness in 21 cases, and invaded 50% or more of the myometrial thickness in 21 cases. The depth of the myometrial invasion was underestimated in nine cases and overestimated in three cases. The staging accuracy with MRI was 74% (33/45 lesions). There was a significant correlation between MR imaging and histopathologic finding in the assessment of myometrial invasion (*p* < 0.001) (Table 1).

According to FIGO staging, 31% patients had stage IA disease, 16% had stage IB disease, 15% had stage II disease. Stage IIIa and IIIc were seen in 7% and 20% patients, respectively. 11% of the patients had IVC disease (Table 2).

In 13 cases (29%), distension of the uterine cavity was present.

The histological grade and disease staging (the depth of myometrial invasion, cervical extension) are predictive of the occurrence of extrauterine spread, pelvic nodal metastases, and impact the prognosis and treatment. Based on the stage of the disease, the treatment options include surgery, radiation therapy and chemotherapy [11,12,13,14].

Cervical extension was noted in eight cases (18%), but was missed on MR imaging in two patients and overstaged in none. Six of them were associated with myometrial invasion in more than 50% of the thickness. There was a significant correlation between MR imaging and histopathologic finding in the assessment of cervical extension (*p* < 0.001) (Table 3).

Regional lymph node invasion was noted in 11 cases (24%). In these patients, the depth of myometrial invasion was bigger than 50% of the thickness in eight cases (73%). In two cases, the myometrial invasion was absent and less than 50% of the thickness, but the histopathology analysis revealed an aggressive histological type of endometrial carcinoma (serous adenocarcinoma and poorly differentiated endometrioid adenocarcinoma G3). One case with reported lymph node invasion and less than 50% myometrial thickness invaded was histologically described as moderately differentiated endometrioid adenocarcinoma G2.

In 36 of the 45 cases, MRI of the lesion showed the characteristics of endometrial cancer and the diagnosis was certain. The main goal of this study is to evaluate the semiology of the endometrial carcinomas that mimic benign lesions and the main factors that may affect the correct diagnosis.

Nine lesions (20%) were described as unequivocal and had unspecific MR appearance. 

On T2-weighted images, the tumors appeared hypointense to the adjacent myometrium in six patients (67%), isointense in two patients (22%) and hyperintense in one patient (11%). Four of the tumors showed high signal intensity on DWI (44%), while six of the tumors showed low signal on the ADC map. The contrast enhancement of the tumors was assessed as low in two cases (22%), intermediate in one case (11%) and heterogenous in six cases (26%). Endometrial thickness was noted in six cases (66%). None of the cases presented distension of the uterine cavity or cervical extension. 

The maximum diameter of the tumor measured maximum 20 mm in three cases (33%), 21 to 40 mm in four cases (45%) 41 to 60 mm in one case (11%) and over 60 mm in one case (11%). 

The histopathologic analysis of these nine cases reported eight cancers as endometrioid adenocarcinomas (four were well differentiated: grade G1; three were moderately differentiated: grade G2; one was poorly differentiated: grade G3) and one cancer as serous adenocarcinoma. All nine patients underwent the classical surgical procedure and the myometrial invasion could be evaluated. On the MR images, eight lesions showed no myometrial invasion, while one lesion invaded more than 50% of the myometrial thickness. The histological examination revealed no myometrial invasion in one case, invasion of less than 50% of the myometrial thickness in five cases and invasion of 50% or more of the myometrial thickness in three cases. The depth of the myometrial invasion was underestimated in seven cases. In eight of the nine cases (89%), the histopathologic report revealed the presence of leiomyomas and two of these cases (22%) were also associated with adenomyosis. The cause of underestimation in these patients was coexisting lesions exhibiting heterogenous intensity and contrast enhancement, which made it difficult to detect the margins of the lesions.

In one of these cases, the MRI examination showed invasion of more than 50% of the myometrial thickness with serous extension, high signal intensity on DWI and low signal on the ADC map, heterogenous structure that suggested and infectious etiology. The result of the histopathological report showed poorly differentiated endometrioid adenocarcinoma G3. 

## 4. Discussion

Patients with noninvasive or locally advanced endometrial carcinoma benefit from surgical treatment. The recent less invasive surgical techniques require an accurate preoperative work-up in order to reduce the risk of understaging the disease and affecting the therapeutic plan.

The age at presentation for endometrial carcinoma has a mean of approximately 63 years, 90% of them present with abnormal vaginal bleeding [15,16].

Compared to other gynecological malignancies, the prognosis of endometrial carcinoma is more favorable, with a 5-year survival rate of 84%. 75% of the patients present with stage I disease [17,18] and the treatment is represented by simple hysterectomy [19].

In our study, 22 (49%) were in the age group between 41 and 60 years, followed by 21 patients (47%) who were more than 60 years old and 2 patients who were less than 40 years old (4%) (27.8%). The most common complaint was postmenopausal vaginal bleeding, seen in 35 patients (78%). The patients in the perimenopausal age group presented with irregular and heavy vaginal bleeding (7 patients, 15%), followed by intermittent spotting (3 patients, 7%). 

The most common histopathology of endometrial carcinoma in our patients was endometrioid adenocarcinoma (38 patients, 85%), 40% of which were grade I, 47% were grade II, 13% were grade III. The next histopathology findings were serous adenocarcinoma (9%), clear cell adenocarcinoma (2%), and mixed adenocarcinoma (4%). A study by Shrivastava et al. [20] also stated that the most of the cases presented with endometrioid adenocarcinoma histology (27 patients, 75%), out of which 48% were grade I, 37% were grade II and 15% were grade III. This was followed by papillary adenocarcinoma, i.e., 22% and adenosquamous carcinoma (3%). A study by Yoney et al. [21] also stated that most of the cases had endometrioid adenocarcinoma histology (227 patients, 92.3%) out of which 51 (61.4%) cases had grade 1 disease.

MR imaging was used in preoperative assessment of the depth of myometrial invasion [22,23,24,25,26], cervical extension [27] and identification of enlarged pelvic lymph nodes [28,29]. In this study, we evaluated the accuracy of MR imaging in diagnosing, staging endometrial carcinoma in order to efficiently plan the treatment of the disease. Endometrial carcinoma could be detected on T2-weighted images as it appeared hypointense to the adjacent myometrium in 16 patients (35%), isointense in 22 patients (49%) and hyperintense in 7 patients (16%). Dynamic MR imagining performed after intravenous of gadolinium chelates is useful in evaluating the tumor due to different vascularity comparing to the myometrium and it helps in differentiating it from the fluid filling the endometrial cavity. The contrast enhancement of the tumors was assessed as low in 30 cases (67%), intermediate in 3 cases (7%) and heterogenous in 12 cases (26%).

The presence and depth of myometrial invasion are important factors in predicting lymph nodes metastases. Patients with myometrial invasion greater than 50% of the thickness have a six to sevenfold increased prevalence of pelvic and lumboaortic lymph node metastases compared with patients with absent or less than 50% myometrial invasion [12]. Planning the extent of lymphadenectomy is determined by the preoperative determination of myometrial invasion.

The presence and depth of myometrial invasion are best assessed on T2-weighted images and appears as an interruption of the junctional zone. However, in postmenopausal women, the junctional zone may be less visible and the myometrium may be thinned due to the involution of the uterus, making the evaluation of myometrial invasion more difficult to assess. In our patients, there was a significant correlation between MR imaging and histopathologic finding in the assessment of myometrial invasion (*p* < 0.001).

Preoperative evaluation of cervical extension is an important step in planning the treatment as it affects the prognosis of the patients. Several studies have shown that macroscopic cervical involvement is associated with a worse prognosis than microscopic involvement and may help in planning radical surgery or additional radiation therapy [30,31]. In this study, cervical infiltration was missed in two patients and overstaged in none. We report an 75% accuracy in detecting cervical involvement. In our patients, there was a significant correlation between MR imaging and histopathologic finding in the assessment of cervical invasion (*p* < 0.001).

Pelvic lymph node status is one of the most important prognosis factors in endometrial carcinoma. MRI may directly depict lymph nodes without contrast medium. MR imaging also has limitations in assessing the status of the lymph nodes, the most important one being differentiating between metastatic and non-metastatic lymph nodes. The presence of central necrosis has a 100% positive predictive value in the diagnosis of metastasis, although it usually occurs when the diameter of the lymph nodes is over 2 cm in size [28]. Another aspect in oncologic patients is setting a cut-off value of the minimal diameter of the lymph node of 1 cm for differentiating metastatic lymph nodes.

In this study, regional lymph node invasion was noted in 11 cases (24%). In these patients, the depth of myometrial invasion was bigger than 50% of the thickness in eight cases (73%). In two cases, the myometrial invasion was absent and less than 50% of the thickness, but the histopathology analysis revealed an aggressive histological type of endometrial carcinoma (serous adenocarcinoma and poorly differentiated endometrioid adenocarcinoma G3). One case with reported lymph node invasion and less than 50% myometrial thickness invaded was histologically described as moderately differentiated endometrioid adenocarcinoma G2.

A limit of this study was represented by the fact that we evaluated only the regional staging of endometrial carcinoma, focusing on the local extension of the tumor and not searching for distant metastases.

## 5. Conclusions

Imaging plays an important role in patients suspected of endometrial cancer, and MRI is considered the imaging method of choice in diagnosing and preoperative staging of endometrial carcinoma. 

Our data confirm the high accuracy of MRI in local staging of endometrial carcinoma, combining the characteristics of the tumor on T2-weighted imaging, diffusion-weighted imaging, ADC mapping, and dynamic contrast administration, which may be particularly helpful in cases of tumors that are either iso- or hyperintense relative to the myometrium.

The future of diagnosing and staging endometrial carcinoma will benefit from upgraded MR protocols that will include MR perfusion, MR spectroscopy, blood oxygen level-dependent MRI and improved contrast agents. These additions will increase the accuracy of the differential diagnosis, offer a better assessment of associated benign and malignant lesions and a more specific characterization of the tumor type in order to aid the surgical protocol and decrease the morbidity and mortality of this neoplasia.

## Figures and Tables

**Table 1:** MRI and histopathologic correlation of myometrial invasion.

			HP_Invasion			Total
			<50%	>50%	without	
MRI_invasion	<50%	Count	12	1	0	13
		Expected Count	6.1	6.1	0.9	13.0
		% within HP_invasion	57.1%	4.8%	0.0%	28.9%
	>50%	Count	3	18	0	21
		Expected Count	9.8	9.8	1.4	21.0
		% within HP_invasion	14.3%	85.7%	0.0%	46.7%
	without	Count	6	2	3	11
		Expected Count	5.1	5.1	0.7	11.0
		% within HP_invasion	28.6%	9.5%	100.0%	24.4%
Total		Count	21	21	3	45
		Expected Count	21.0	21.0	3.0	45.0
		% within HP_invasion	100.0%	100.0%	100.0%	100.0%
**Chi-Square Tests**						
			Value		df	*p* value
Pearson Chi-Square			32.946		4	<0.001
Likelihood Ratio			34.104		4	<0.001
N of Valid Cases			45			

**Table 2:** FIGO staging of the patients included in this study.

Figo Staging	Number of Patients	Percentage
IA	14	31
IB	7	16
IC	0	0
IIA	0	0
IIB	2	4
IIC	5	11
IIIA	3	7
IIIB	0	0
IIIC	9	20
IVA	0	0
IVB	0	0
IVC	5	11

**Table 3:** MRI and histopathologic correlation of cervical extension.

						HP Cervix Invasion				Total
						0		1		
MRI cervix invasion	0		Count			37		2		39
			Expected Count			32.1		6.9		39.0
			% within HP cervix invasion			100.0%		25.0%		86.7%
	1		Count			0		6		6
			Expected Count			4.9		1.1		6.0
			% within HP cervix invasion			0.0%		75.0%		13.3%
Total			Count			37		8		45
			Expected Count			37.0		8.0		45.0
			% within HP cervix invasion			100.0%		100.0%		100.0%
**Chi-Square Tests**										
		Value		df	Asymptotic Significance (2 sided)		Exact Sig. (2 sided)		Exact Sig. (1 sided)	
Pearson Chi-Square		32.019		1	<0.001					
Continuity Correction		25.858		1	<0.001					
Likelihood Ratio		26.343		1	<0.001					
Fisher’s Exact Test							<0.001		<0.001	
Linear-by-Linear Association		31.308		1	<0.001					
N of Valid Cases		45								

## Data Availability

Data are available upon request from the corresponding authors.
